# Diabetes, cardiovascular disease and the microcirculation

**DOI:** 10.1186/s12933-018-0703-2

**Published:** 2018-04-18

**Authors:** W. David Strain, P. M. Paldánius

**Affiliations:** 1 Diabetes and Vascular Medicine Research Centre, NIHR Exeter Clinical Research Facility and Institute of Biomedical and Clinical Science, University of Exeter Medical School, Royal Devon & Exeter NHS Foundation Trust, Barrack Road, Exeter, EX2 5AX UK; 2 Novartis Pharma AG, Basel, Switzerland

**Keywords:** Microcirculation, Type 2 diabetes mellitus, Hypertension, Cardiovascular disease, Microvascular changes, Microalbuminuria

## Abstract

Cardiovascular disease (CVD) is the leading cause of mortality in people with type 2 diabetes mellitus (T2DM), yet a significant proportion of the disease burden cannot be accounted for by conventional cardiovascular risk factors. Hypertension occurs in majority of people with T2DM, which is substantially more frequent than would be anticipated based on general population samples. The impact of hypertension is considerably higher in people with diabetes than it is in the general population, suggesting either an increased sensitivity to its effect or a confounding underlying aetiopathogenic mechanism of hypertension associated with CVD within diabetes. In this contribution, we aim to review the changes observed in the vascular tree in people with T2DM compared to the general population, the effects of established anti-diabetes drugs on microvascular outcomes, and explore the hypotheses to account for common causalities of the increased prevalence of CVD and hypertension in people with T2DM.

## Background

Type 2 diabetes mellitus (T2DM) and hypertension are established risk factors for cardiovascular disease (CVD), and people with T2DM and hypertension have an increased risk of cardiovascular (CV) mortality compared with those with either condition alone [1]. This excess risk is suggested to be due to the synergistic effect on large and small blood vessels simultaneously, thereby reducing the potential for compensatory collateralization protecting organs from the adverse consequences of damage to either vascular bed. The principle role of the vasculature is to deliver oxygen and nutrients to the tissues—whether that is the heart, the brain, or the kidney. The functional changes occurring in T2DM and hypertensive conditions significantly alter the haemodynamic stress on the heart and other organs. However, the different physiology, mechanisms and changes at the microvascular level differ from those at the macrovascular level in T2DM and hypertension, which in turn have significant implications with respect to future CV risk.

### Vascular anatomy in cardiovascular disease

**Fig. 1:**
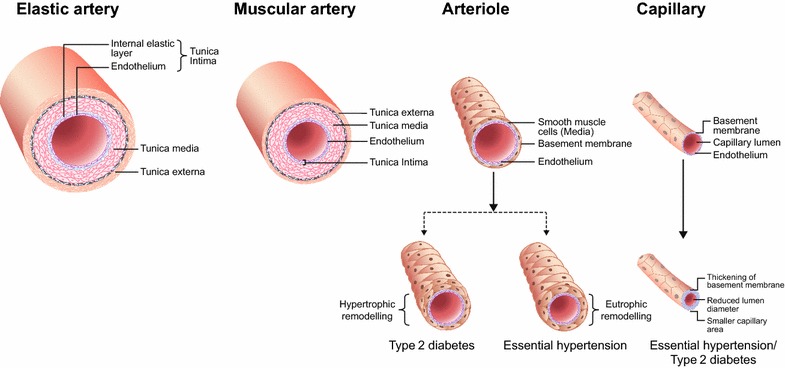
Structural hierarchy of arterial tree in health and disease conditions

Although there is increasing evidence that the venous tree regulates cardiac output and total body circulating fluid, the majority of the pathology occurs within the arterial circulation. Broadly, the arterial tree spanning from the large coronary artery to the minute capillaries is comprised of four components—elastic (conduit) arteries, muscular conduit arteries, muscular resistance arterioles and capillaries—each representing a distinct vessel system (Fig. 1) with a distinct role to play in the circulation [2]. Elastin and collagen, the major structural proteins of elastic and muscular conduit arteries, respectively, provide mechanical strength to the vessel wall for the conduct of blood from the heart to peripheral organs [3]. Their abundance along the longitudinal aortic axis is largely determined during the developmental stage and remains quite stable after that, due to the extremely low turnover [2]. The basic architecture of the arterial tree displays a progressive change from predominantly elastin and vascular smooth cells at the aortic arch, gradually giving way to a collagen rich media by the distal aorta (Table 1). Over the last five centimetres of the thoracic aorta and aortic branches, there is a rapid transition to a predominantly collagen and vascular smooth cell muscular artery. In the resistance arterioles and capillaries, vascular smooth muscle (VSM) cells become increasingly sparse until these are no more than one cell layer in the terminal branches. VSM cells have differing embryonic origins in the vessel beds, with proximal elastic and muscular vessels derived from ectodermal tissue, whereas small muscle beds and arterioles have mesodermal origin. Thereby, the formation of microcirculation is a result of the complex process of angiogenesis from these mesodermal tissues which takes place during embryonic development as well as during adulthood (e.g. during hypoxic conditions) [2]. These differences in embryology have potential pharmacological and clinical consequences later in life as they are thought to trigger differential effects of certain classes of vasodilators such as calcium channel blockers or α-adrenoceptor antagonists on proximal versus distal VSM cells.

**Table 1:** Characteristics of components of the arterial tree

	Elastic arteries	Muscular conduit arteries	Muscular resistance arterioles	Capillaries
Diameter	> 2 mm	150 µm–2 mm	8–150 µm	< 8 µm
Regulation	Media structure > endothelium	Media structure and endothelium	Endothelium > media structure	Endothelium only
Function	Conduit: elastic recoil (diastolic BP)	Conduit: minor resistance	Resistance	Nutrient and waste exchange

*BP* blood pressure

### Hypertensive target organ damage in people with diabetes

One of the hallmarks of hypertensive vascular damage is increased arterial stiffness in the large elastic arteries [4]. Arterial stiffness contributes to the pathogenesis of atherosclerosis and independently predicts CV death after adjustment for hypertension, age and gender in patients with end stage renal failure [4], essential hypertension [5] and T2DM [6]. Greater arterial stiffness [7] and vascular endothelial cell dysfunction [8] were reported in patients with T2DM. Concomitant T2DM and hypertension is also associated with greater arterial stiffness than either condition alone, independent of conventional CV risk factors such as gender, smoking history and ethnicity [9, 10]. Furthermore, in people with diabetes, the cell types which maintain integrity of the vascular wall in the macrocirculation are more prone to damage, particularly in the presence of CV risk factors [11]. These macrovascular changes, however, are evident in the pre-diabetic and pre-hypertensive stages, raising the possibility of a vascular aetiology in the pathogenesis of diabetes and hypertension [12, 13].

Several mechanisms have been proposed to account for the greater arterial stiffness in patients with T2DM and hypertension. Elevated glycaemia is a major determinant of both arterial stiffness and carotid intimal media thickness (IMT), the latter of which is another well-established measure of blood pressure (BP)-related damage independently predictive of CV events [14, 15]. Chronic hyperglycaemia is known to be associated with the build-up of advanced glycation end-products (AGEs), which, lead to arteriosclerosis [16]. This could account for the impact of glycaemia on endothelial function. A meta-analysis reported that an increase in carotid IMT by 0.13 mm is associated with an increase in CV risk by nearly 40% in patients with T2DM compared with control subjects [17].

Oxidative stress is an alternative mechanism which has been suggested to exacerbate macrovascular damage in patients with diabetes. Reactive oxygen species (ROS) can be induced by multiple biochemical pathways including activation of the polyol pathway and the non-enzymatic formation of AGEs [16, 18, 19], each of which could damage the endothelial system. Supportive evidence includes the observation that anti-oxidant drugs inhibit the pathological neovascularisation of endothelial cells by attenuating the production of these ROS under hyperglycaemic conditions [20]. An alternative, possibly complementary, mechanism of vascular damage is the inactivation or suppression of nitric oxide (NO) by oxygen-derived free radicals; interestingly this has been associated with glycaemic variability rather than by glycaemia itself [21]. This observation is supported by the association between glycaemic variability, as measured by mean amplitude of glycaemic excursion (MAGE), and clinically relevant outcomes [22]. Glycaemic variability has been shown to be a strong prognostic factor for poorer cardiac outcomes in subjects with T2DM after acute myocardial infarction, supplanting other established measures of glycaemia, including glycated haemoglobin (HbA1c), fasting plasma glucose or postprandial glucose alone [23]. The use of dipeptidyl peptidase-4 (DPP-4) inhibitors to reduce daily glucose fluctuations has been associated with a reduction in oxidative stress and inflammation [24]: within a 3-month period, reduction in glycaemic variability caused a commensurate and proportionate reduction in carotid IMT [25], suggesting that glycaemic variability could be a potentially reversible early therapeutic target to partially address the increased CVD risk in those with T2DM.

Further, in chronic vascular conditions, the incidence of macrovascular events is usually accompanied by significant and progressive microvascular pathological impediments and dysfunction. The effects of increasing peripheral vascular disease (PVD) risk on indices of microvascular dysfunction confirm presence of multiple predictors of microvasculopathy and health outcomes of macrovascular events: studies of skeletal muscle microcirculation in rat models indicate greater heterogeneity in perfusion distribution and reduced flexibility in microvascular network, progressive decrease in NO bioavailability, arachidonic acid metabolism, as well as myogenic activation and adrenergic constriction [26].

### The role of microcirculation is universal

The emphasis on large vessel diseases such as increased arterial stiffness and carotid IMT ignores the contribution of the microcirculation to CVD. Whilst the association between disease of the conduit or resistance arteries and CVD has been explored and well-characterised, much of the variance in the increased frequency but also clinical symptoms of CVD in diabetes remains unexplained. For example, in patients with heart failure (HF), the presence of diabetes increases the risk of longer hospital stays, recurrent HF hospitalisations and mortality in comparison with patients without diabetes [27–29]. The lack of association between hyperinsulinemia and insulin resistance in microvascular dysfunction was questioned in the past [30] but it is now well-established that endothelial dysfunction of microvascular origin, in the absence of obstructive epicardial coronary disease, such as myocardial ischemia due to coronary stenosis may lead to clinical manifestation and symptoms indicative of microvascular angina even at rest [31]. However, the role of overall, conventionally assessed, improved glycaemic control on microvascular function is unclear [32]. Therefore, although AGEs or persistent insulin resistance are speculated to cause progressive haemodynamic dysfunction and increased CV events in patients with diabetes [28], the exact mechanisms associating hypertension and atherosclerosis in the background of diabetes are not clearly understood. Yet the aspect of microcirculatory function once unfolded may lead to the development of future novel therapeutic targets especially in subjects with diabetes.

The microcirculation is a network of blood vessels < 150 µm in diameter, comprising arterioles, capillaries and venules. This network is responsible for the primary function of the vascular tree and regulation of tissue perfusion for optimal exchange of gases and removal of metabolic waste products [33] and may contribute to the unexplained variance in the association between T2DM and hypertension. There are significant differences in the way small arteries remodel in response to hypertension in people with or without T2DM. In patients with essential hypertension alone, the media-to-lumen ratio of small arteries is increased due to reduced lumen and external diameter and greater media thickness, with minimum changes in the total amount of wall tissue (Fig. 1) [34]. These structural alterations in small arteries due to inward eutrophic remodelling without net cell growth, result in decreased vasodilator reserves and changes in the distensibility of arterioles [35, 36]. On the contrary, in patients with T2DM, the media cross-sectional area of small vessels is increased, suggesting hypertrophic remodelling [37]. The mechanisms underlying hypertrophic remodelling may include increased wall stress due to an impaired myogenic response of small arteries in T2DM [38]. The manifestation of endothelial dysfunction in T2DM may be related to increased microvascular permeability to large molecules, such as albumin [39]. Furthermore, in T2DM, vascular dysfunction at the capillary network can alter the insulin delivery and thus, the impaired insulin sensitivity [40]. Linking these observations, impaired microvascular auto-regulatory myogenic responses in populations with T2DM predicts urinary albumin excretion rate (UAER), and accounts for its association with adverse cardiac remodelling [41, 42]. Finally, alterations in the vascular extracellular matrix (increased collagen-to-elastin ratio) are observed in the vessel wall of people with T2DM [37], probably due to inflammatory and pro-fibrotic changes. A recent population-based study reported no difference in wall thickness and cross-sectional area of retinal arterioles between healthy, T2DM and hypertensive subjects at early stages of disease but greater wall thickness in subjects with a diabetes duration of > 60 months compared with other groups, suggesting hypertrophic remodelling in T2DM with advancing disease duration [43].

Small arterioles and capillaries also exhibit differential vascular remodelling in response to hypertension and T2DM. The number of vessels perfused in the vascular bed and the arteriolar diameter determine the peripheral vascular resistance. Microvascular rarefaction can be due to the presence of a reduced number of perfused vessels in the vascular bed (functional rarefaction) or reduced number of vessels in the tissue (structural rarefaction) [44]. In most vascular beds, at a given time, only a fraction of microvessels are perfused, and the non-perfused/reserved vessels are called upon during high metabolic demand. Structural loss of vessels may follow progressive non-perfusion. In patients with hypertension and T2DM, rarefaction has been consistently reported in myocardial microvessels, resulting in a reduced coronary flow reserve. Also, maximal blood flow reduces due to structural abnormalities in the coronary microcirculation and/or functional factors such as endothelial dysfunction, or systemic inflammation [44–46]. Although not associated with atherosclerosis, this predicts cardiac symptoms, and may explain the high prevalence of refractory [47] and microvascular angina [31], especially in people with diabetes, despite normal or only mildly diseased coronary arteries.

### Microcirculatory dysfunction: cause or effect?

The microcirculatory changes noted in the retinal and renal systems have been extensively studied to understand the predictive role of glycaemic variations early in diabetes [48]. Diabetic retinopathy, the leading cause of premature blindness among patients with T2DM [49], is linked to an increased risk of CV mortality [50]. Changes in the retinal microvasculature of healthy individuals are independently associated with future risk of T2DM [51] as well as congestive HF and CV mortality [52, 53], suggesting a microvascular aetiology in the pathogenesis of T2DM. Conversely, in patients with T2DM, before the onset of retinopathy, regional differences in retinal metabolic changes are reported, without an associated regional variance of microvascular haemodynamics [54]. These studies confirm an association between development and progression of the microvascular disease to macrovascular disease—however, the nature of association and direction of causal effect has not been established. Studies assessing nephropathy, atherosclerosis and metabolic syndrome may provide additional evidence to support this.

### The effect of anti-diabetes drugs on microcirculation

The direct effects of anti-diabetes agents on vascular structure and function have been studied using different microvascular models in short-term studies [55–57]. The effects of glucagon-like peptide-1 (GLP-1)-based therapies on microvasculature are heterogeneous. The addition of liraglutide in patients with T2DM showed either amelioration in the microvascular hyperaemic response [55] or no effect on peripheral endothelial function [58]. Treatment with a DPP-4 inhibitor improved microvascular function with increased hyperaemia area, and resting and peak blood flow in the fasting state [59]. In patients with T2DM, saxagliptin treatment normalised the retinal capillary flow [56], whereas vildagliptin showed improved retinal microvascular blood flow beyond glucose control [57]. Overall, although experimental studies reveal early beneficial effects of DPP-4 inhibitors and GLP-1 agonists on diabetic microvascular complications, clinical data regarding the direct effects of these classes of drugs on microangiopathy, independent of glucose control, is insufficient and warrants additional studies for confirmation. A recent 12-week, randomised controlled trial in patients with T2DM concluded that beneficial effects of GLP-1-based therapies on glycaemic control and BP are not mediated through microvascular changes [33], suggesting further investigation of GLP-1 agonists’ effects on microcirculation. The LEADER study, using liraglutide, was associated with improvements in microvascular function beyond the benefit anticipated from epidemiological models [60].

In light of the CV safety concerns with certain anti-diabetes drugs [61], the Food and Drug Association (FDA) and the European Medicines Agency (EMA) mandated demonstration of CV safety of new anti-diabetes drugs as part of the approval process [62, 63]. Owing to the large enrolled population and longer follow-up duration, evidence from these trials or qualified meta-analyses can be used to assess the effects of anti-diabetes drugs on microcirculation. Interestingly, in the SUSTAIN-6 and LEADER trials, the GLP-1 agonists semaglutide and liraglutide, respectively, reduced the incidence of nephropathy [60, 64]—these benefits were not observed with the exendin-4 based lixisenatide and exenatide in their respective CV outcome trials [65, 66]. This observed heterogeneous response may represent differences in the effect of the agents or the selected population; the ELIXA study enrolled patients with T2DM who experienced an acute coronary syndrome, and all studies included individuals with long-term T2DM with established CVD. However, one potential explanation could be a GLP-1 receptor independent effect of the GLP-1 analogue that is not mirrored with the exendin-4 derivatives [67]. Paradoxically, the LEADER and SUSTAIN-6 studies also demonstrated an increased incidence of retinopathy-related events [60, 64]—suggested to be due to the dramatic reduction in HbA1c in the early phases of these trials with osmotic shifts that have been well-characterised in trials of other agents, including sulphonylureas and insulins. Further, the results are limited by the binary outcome of retinopathy, whereas we know that retinopathy is itself a dynamic process that changes with glycaemic control.

Evidence from the EMPA-REG and CANVAS trials with the sodium–glucose cotransporter 2 (SGLT-2) inhibitors empagliflozin and canagliflozin, respectively revealed inconsistent microvascular outcomes in patients with T2DM and high CVD risk [68–70]. Although both agents demonstrated a favourable effect on selected renal outcomes, a higher risk of amputation in toes, feet, or legs was observed in patients treated with canagliflozin [69, 70]. This might probably be due to the impairment in the capillary network perfusion in the lower extremities of these patients with established microvascular complications. However, unlike empagliflozin and canagliflozin, short term treatment with dapagliflozin reduced retinal capillary flow and stabilised early structural remodelling (in arteriolar wall cross-sectional area and wall-to-lumen ratio) in patients with T2DM [71]. The understanding on mechanism of action of SGLT-2 inhibitors on microcirculatory as well as macrovascular changes is limited and further research is warranted to explore the above findings. These varying responses in microvascular outcomes in different vascular beds suggest direct effects of anti-diabetes agents on the target organs.

In addition to anti-diabetes agents, statins were reported to improve endothelial dysfunction and microvascular reactivity in patients with T2DM and dyslipidaemia, suggesting positive outcomes on CV morbidity and mortality of these class of drugs [72].

### Vascular endothelial growth factor and microcirculation

Vascular endothelial growth factor (VEGF) stimulates angiogenesis and can affect the microvascular structure and function in patients with T2DM and hypertension [73]. In patients with diabetic macular oedema, anti-VEGF therapy attenuates the progression of angiogenesis in retinal microvasculature by acting on endothelial cells, which affects the systemic microcirculation as well. Treatment with bevacizumab for 6 months in patients with metastatic colorectal cancer resulted in reduced endothelial dysfunction and capillary rarefaction, as assessed by reduction of mean dermal capillary density and vasodilation in the dorsum of the fingers [74]. Therefore, similar to patients with essential hypertension, bevacizumab can cause increased systemic vascular resistance as a result of microvascular rarefaction. Similarly, an intravitreal injection of ranibizumab ameliorated vision in patients with macular oedema owing to branch retinal vein occlusion by reducing the width and relative flow volume in retinal arteries and veins [75].

### Microalbuminuria: from epidemiology to clinical practice and back again

Clifford Wilson and Paul Kimmelstiel for the first time in 1936, described UAER as a feature of glomerulosclerosis with poor prognosis. Since then, the role of UAER has evolved from a marker of renal microcirculation to a predictor of a host of circulatory defects. Several epidemiological studies reported elevated UAER as a predictor of future CV events and mortality in diabetes, renal failure, hypertension and the general population [76–78]. UAER also predicts survival after myocardial infarction [79] and stroke [80]. Therefore, UAER or its biochemical equivalent, albumin:creatinine ratio (ACR), has been widely used as a surrogate marker for assessing microcirculatory target organ damage in patients with T2DM. However, the minimum threshold level of albuminuria as a prognostic indicator of microcirculatory defects is still debated, since an association has also been observed even below the physiological levels that can be measured using commercial kits [81]. A link between albuminuria levels and increased risk of microcirculatory defects/CVD events (odds ratio [OR] 1.20, 95% confidence interval [CI] 1.08–1.33) was reported at values ≥ 10.5 mg/24 h in patients with T2DM [82].

Increased haemodynamic stress and vascular permeability to macromolecules in diabetes can lead to adverse CV events [83]. However, in the absence of a clear mechanistic pathway linking microalbuminuria to adverse CV outcomes, many clinicians consider it as a marker of blood pressure exposure. Nevertheless recent mechanistic studies suggest that the systemic microvascular disturbances that account for the association between microalbuminuria and cardiac target organ damage are independent of either acute or long term BP effects [41, 42].

### Microvascular function as an aetiopathogenic step in those with diabetes and CVD

Patients with T2DM alone are at a higher risk of CV events and CV mortality compared with those without diabetes [84]. The presence of T2DM has a similar impact on morbidity and mortality as the history of CV event [85]. Structural microvascular damage precedes the development of CV events in patients with T2DM whereas changes in microvascular function occur before microangiopathy [86]. In patients with type 1 diabetes mellitus (T1DM), microvascular defects develop several years after diagnosis, probably relative to glycaemic control [87]. On the other hand, in women with a history of gestational diabetes [88] and in those at risk of developing T2DM [89, 90], microvascular defects are manifested from the stage of diagnosis. Therefore, the fact that an increase in microvascular disease (diabetic retinopathy) defines a diagnostic cut-off value for HbA1c indicates the presence of early and progressive pathophysiological defects even prior to the confirmatory diagnosis based on glycaemia.

The link between microangiopathy and functional microvascular alterations in T2DM and their association with good glycaemic control has been strengthened by studies on skin microvascular hyperaemic responsiveness [91–93]. The degree of glycaemic control (percentage decrease in HbA1c over a 12-month period) was strongly associated with percentage improvement in maximum hyperaemic response (r^2^ = 0.53, p = 0.004), suggesting that early microvascular changes in T2DM are potentially reversible with glycaemic control [91]. Recent findings suggest that early good glycaemic control is associated with improved microvascular function in patients with T2DM and CVD, but lost in those with prolonged disease, suggesting early, initial aggressive glycaemic control to delay/prevent microvascular complications even in patients with co-morbid conditions (CVD) [94]. However, the association between good glycaemic control and microvascular function may not correlate with an improvement in CVD event rates. Rosiglitazone, a peroxisome proliferator-activated receptor gamma antagonist, improved NO-dependent vascular response in the skin micro-vessels of patients with T2DM independent of glycaemic changes [95], but resulted in a concomitant increase in the CV event rate [61]. Interestingly, additional studies with rosiglitazone indicated an increase in the risk of myocardial infarction while it ameliorated the risk of stroke.

The relationship between CV risk and microcirculatory function has been explored by studying the skin microcirculatory reactivity. In patients with an increased risk of coronary heart disease (CHD), a strong association between skin microvascular function (capillary recruitment and impaired endothelium-dependent vasodilation) and 10-year CHD risk scores (calculated from the Framingham risk scores), independent of gender and body mass index was observed [96]. Additional studies assessing the link between skin microvascular function and risk of CV events supported an association between impaired systemic microvascular responses in patients with angiographically confirmed atherosclerotic coronary artery disease (CAD) [97, 98]. Despite the clear attenuation in microvascular response in patients with CAD compared with healthy controls, a direct association between atherosclerotic burden and impaired systemic microcirculation could not be established, suggesting the association between CVD and microcirculatory function is more complex than assumed. Similar findings are supported by a study which showed how intensified insulin treatment in subjects with T1DM was associated with improvement in skin microcirculation versus standard insulin treatment, leading to lower incidence of ischaemic foot ulcers [99]. The role of chronic hyperglycaemia in insulin-dependent subjects, on the relationship between endothelial-dependent skin vasodilation was associated with HbA1c only and demonstrated the lack of direct effect of pre-existing severe micro- or macrovascular risk factors or complications with induction in skin microcirculation and blood flow.

The ethnic variations in microcirculatory function reported in studies with European and African-Caribbean populations further highlight the complex interplay between microvascular function and CV disease. African-Caribbean people with T2DM, who have a low risk of heart disease despite increased prevalence of salt sensitive hypertension, diabetes and insulin resistance compared to their European counterparts [100], are expected to have better systemic microcirculatory responses. On the contrary, a study on ethnic differences in microvasculature demonstrated impaired microvascular structure and function in the general African-Caribbean population compared with Europeans [101], which was further impaired in patients with T2DM and was unexplained by conventional CV risk factors [102]. The attenuation of microvascular function re-confirms the increased risk of renal disease [103] and retinopathy in African-Caribbean people [104] among other population-based studies. Furthermore, our current understanding that impaired macrovascular function at different vascular beds follows the same mechanism is challenged by contrasting observations; there is a higher incidence of stroke and HF and relative protection from atherosclerotic disease [100] in African-Caribbean patients compared with Europeans. It also supports the role of microcirculatory dysfunction in the aetiopathogenesis of stroke. This is further supported by data from the atherosclerosis risk in communities (ARIC) study, wherein microvascular damage assessed by retinopathy and cerebral white matter lesions predicted the risk of future macrovascular dysfunction (stroke) [105]. Similarly, elevated UAER, a marker of systemic microcirculatory dysfunction, predicts the risk of incident stroke and survival after stroke [80].

### Microcirculation and clinical practice

Patients with a low to moderate absolute risk report a projected high incidence of CVD and therefore need clinical intervention. These decisions, however, are primarily based on the likelihood of a CV event rather than complete assessment of an individual risk of developing a CV event. Knowledge regarding structural and functional alterations in different microvascular beds in subjects with co-existing T2DM and hypertension, and their relationship with macrovascular diseases, can be utilised for clinical decision-making. Retinal microvasculature is a simple model to investigate in subjects with T2DM and can be utilised on a large-scale for translation into clinical practice. Skin microvasculature is another accessible model to investigate diabetes-associated microvascular complications [106, 107].

Similarly, the ease of measuring ACR using a single urine specimen qualifies UAER as a tool for estimating the future risk of CV events, which can be translated into clinical practice [108]. Therefore, relapse or early prevention of progression of urinary albumin excretion should be considered a clinical target to reduce the risk for CVDs. However, large-scale studies evaluating the long-term cost-effectiveness of using UAER as a screening and therapeutic outcome measure to manage CVD in high risk patients (i.e., those with hypertension, history of stroke, transient ischaemic attack, myocardial infarction, and diabetes) are required for verification of widespread clinical investigations.

## Conclusions

Over the past few decades, epidemiological studies have elucidated the role of impaired microcirculation in people with diabetes and aetiopathogenesis of CVD. This has led to the recognition of the prevalence of microvascular disease. Furthermore, the prognostic value of incidence of microvascular disease in predicting CVD is now acknowledged. The focus of present-day epidemiological studies is to understand the association between pathological mechanisms and the risk factors to ascertain whether they are targets of therapeutic value or risk markers of CVD. These studies have contributed to the evidentiary framework in favour of clinical monitoring of microvascular function, and spurred the initiation of mechanistic studies by redefining our knowledge of vascular disease, particularly in people with diabetes.

## Abbreviations

ACR: albumin:creatinine ratio
AGEs: advanced glycation end-products
BP: blood pressure
CAD: coronary artery disease
CHD: coronary heart disease
CI: confidence interval
CV: cardiovascular
CVD: cardiovascular disease
DPP-4: dipeptidyl peptidase-4
EMA: European Medicines Agency
FDA: Food and Drug Association
GLP-1: glucagon-like peptide-1
HbA1c: glycated haemoglobin
HF: heart failure
IMT: intimal medial thickness
MAGE: mean amplitude of glycaemic excursion
NO: nitric oxide
OR: odds ratio
ROS: reactive oxygen species
T2DM: type 2 diabetes mellitus
UAER: urinary albumin excretion rate
VEGF: vascular endothelial growth factor
VSM: vascular smooth muscle
