# Correction to: Diabetes, cardiovascular disease and the microcirculation

**DOI:** 10.1186/s12933-021-01307-5

**Published:** 2021-06-11

**Authors:** W. David Strain, P. M. Paldánius

**Affiliations:** 1 Diabetes and Vascular Research Centre, Institute of Biomedical and Clinical Science, University of Exeter Medical School, Royal Devon & Exeter NHS Foundation Trust, Barrack Road, Exeter, UK; 2 Novartis Pharma AG, Basel, Switzerland; 3 Research Group for Clinical and Molecular Metabolism (CAMM), Children’s Hospital, Helsinki University Hospital (HUS), Helsinki University, Helsinki, Finland

## Correction to: Cardiovasc Diabetol (2018) 17:57 https://doi.org/10.1186/s12933-018–0703-2

Following publication of the original article [1], it has been brought to our attention that the article in Ref. [72] was retracted in March 2019.

The authors suggested deleting the following statement in section ‘The effect of anti‑diabetes drugs on microcirculation’ from the original publication, citing the retracted article. The overall conclusions of the review article are not affected by this erratum.

“In addition to anti-diabetes agents, statins were reported to improve endothelial dysfunction and microvascular reactivity in patients with T2DM and dyslipidaemia, suggesting positive outcomes on CV morbidity and mortality of these class of drugs [72].”

Furthermore, the affiliation of author Päivi Maria Paldánius has changed since the original publication. The updated affiliations are provided with this erratum.
